# A Robust Image Registration Interface for Large Volume Brain Atlas

**DOI:** 10.1038/s41598-020-59042-y

**Published:** 2020-02-07

**Authors:** Hong Ni, Chaozhen Tan, Zhao Feng, Shangbin Chen, Zoutao Zhang, Wenwei Li, Yue Guan, Hui Gong, Qingming Luo, Anan Li

**Affiliations:** 1 Britton Chance Center for Biomedical Photonics, Wuhan National Laboratory for Optoelectronics, Huazhong University of Science and Technology, Wuhan, China; 2 MoE Key Laboratory for Biomedical Photonics, School of Engineering Sciences, Huazhong University of Science and Technology, Wuhan, China; 3 HUST-Suzhou Institute for Brainsmatics, JITRI Institute for Brainsmatics, Suzhou, China

**Keywords:** Neural circuits, Scientific data, Image processing, Neural circuits, Image processing

## Abstract

Accurately mapping brain structures in three-dimensions is critical for an in-depth understanding of brain functions. Using the brain atlas as a hub, mapping detected datasets into a standard brain space enables efficient use of various datasets. However, because of the heterogeneous and nonuniform brain structure characteristics at the cellular level introduced by recently developed high-resolution whole-brain microscopy techniques, it is difficult to apply a single standard to robust registration of various large-volume datasets. In this study, we propose a robust Brain Spatial Mapping Interface (BrainsMapi) to address the registration of large-volume datasets by introducing extracted anatomically invariant regional features and a large-volume data transformation method. By performing validation on model data and biological images, BrainsMapi achieves accurate registration on intramodal, individual, and multimodality datasets and can also complete the registration of large-volume datasets (approximately 20 TB) within 1 day. In addition, it can register and integrate unregistered vectorized datasets into a common brain space. BrainsMapi will facilitate the comparison, reuse and integration of a variety of brain datasets.

## Introduction

Mapping brain structures in three dimensions is necessary to thoroughly understand brain functions^1^. Creating a comprehensive space in which various brains are mapped encompasses complex spatiotemporal information that can greatly facilitate the comparison^2^, reuse^3^ and integration^4^ of brain datasets. Drawing a stereotaxic brain atlas^5,6^ provides a unified spatial reference for addressing this issue. However, with the rapid development of high-resolution whole-brain microscopic imaging^7–9^, the obvious heterogeneous and nonuniform^10^ characteristics of brain structures at the cellular level make it difficult to map the variety of experimental datasets from different individuals and modalities to a standard brain space using the uniform registration methods^11^ that have been used effectively in previous macroscopic datasets, such as magnetic resonance imaging (MRI) datasets. In addition, given the large-volume datasets produced during imaging, we urgently need a robust nonlinear registration pipeline that can register massive spatial information datasets with cellular resolution.

Previous studies^11–15^ have been conducted to solve the registration of whole-brain three-dimensional datasets, especially within the MRI field. Among these, gray-level-based registration algorithms^11^ can effectively achieve nonlinear registration of MRI datasets with uniform signals. These methods can also be applied to optical microscopic images. For example, Leonard *et al*.^16^ processed serial two-photon (STP) datasets to obtain an average brain. However, the gray-level-based methods are highly dependent on the quality of the original image signals. Similarly optical microscopy images are susceptible to variations in sample preparation and imaging processes. Hence, for the more complex optical microscopy images, feature-based registration algorithms^17–19^ are the better choice because the factors that affect registration accuracy can be changed from the grayscale level to the extracted features level; then, the bottleneck involves accurately and objectively extracting sufficient features. Ohnishi *et al*.^14^ completed the registration of two-dimensional microscopy images to MRI data by manually extracting feature points, but their number was limited and subjective. Wang *et al*.^20^ used automatic methods to extract feature points and achieved the registration of histopathological data, but the accuracy of the automatic recognition methods used was greatly affected by image quality. Fürth *et al*.^17^ combined the benefits of artificial and automatic methods and achieved the registration of two-dimensional continuous microscopy images to a brain atlas. Briefly, when neuroscientists are struggling to obtain a valuable experimental dataset, they often find that it is difficult to extract sufficient feature points accurately to ensure the registration quality.

Another challenge occurs with the TB-scale large-volume whole-brain datasets brought by imaging at the cellular level. Generally, the registration tools such as ITK that are widely used in biomedical fields are suitable for the registration of GB-scale data volumes. For example, in one study^21^ registering a 12.5 μm^3^ resolution whole-brain dataset approximate to 1 GB. Clearly, the nonlinear registration algorithm is a global optimization solution that consumes large amounts of memory^22^. Thus, the strategy of obtaining the transformation parameters at low resolution and warping massive amounts of data in blocks is a better choice. Qu L. *et al*.^23^ developed a block-based fast warping tool that successfully aligned several GB-volume datasets. However, TB-scale whole-brain datasets introduce more serious computing and efficiency problems, making it difficult to achieve quick registration for TB-scale large-volume datasets by simply stacking hardware.

Here, we propose BrainsMapi, a novel registration interface for quickly and accurately mapping three-dimensional brain image datasets at the cellular level to the standard brain atlas. It can process various intramodal datasets, individual datasets with large-differences, and multimodal datasets. We used BrainsMapi to register four types of datasets (MRI^24^, Nissl staining (Micro-Optical Sectioning Tomography, MOST)^7^, propidium iodide (PI) staining (Brain-wide Precision Imaging system, BPS)^8^ and STP^9^) to the Allen Common Coordinate Framework version 3 (Allen CCFv3)^6,16^ as a robust and accurate demonstration. The registration results are highly accurate at the brain region level (Dice score > 0.9), and there is no significant difference between registration results and manual results in the identifiable nuclei level at a 10 μm resolution (P > 0.05). Furthermore, BrainsMapi can also process large-volume three-dimensional brain image datasets: we demonstrate a quick registration of whole brain datasets consisting of approximately 20 teravoxels. In addition, BrainsMapi can align labeled data from an original unregistered dataset, which allows the integration of datasets comprising neuroscience information from different sources into a common brain space.

## Materials and Methods

**Figure 1:**
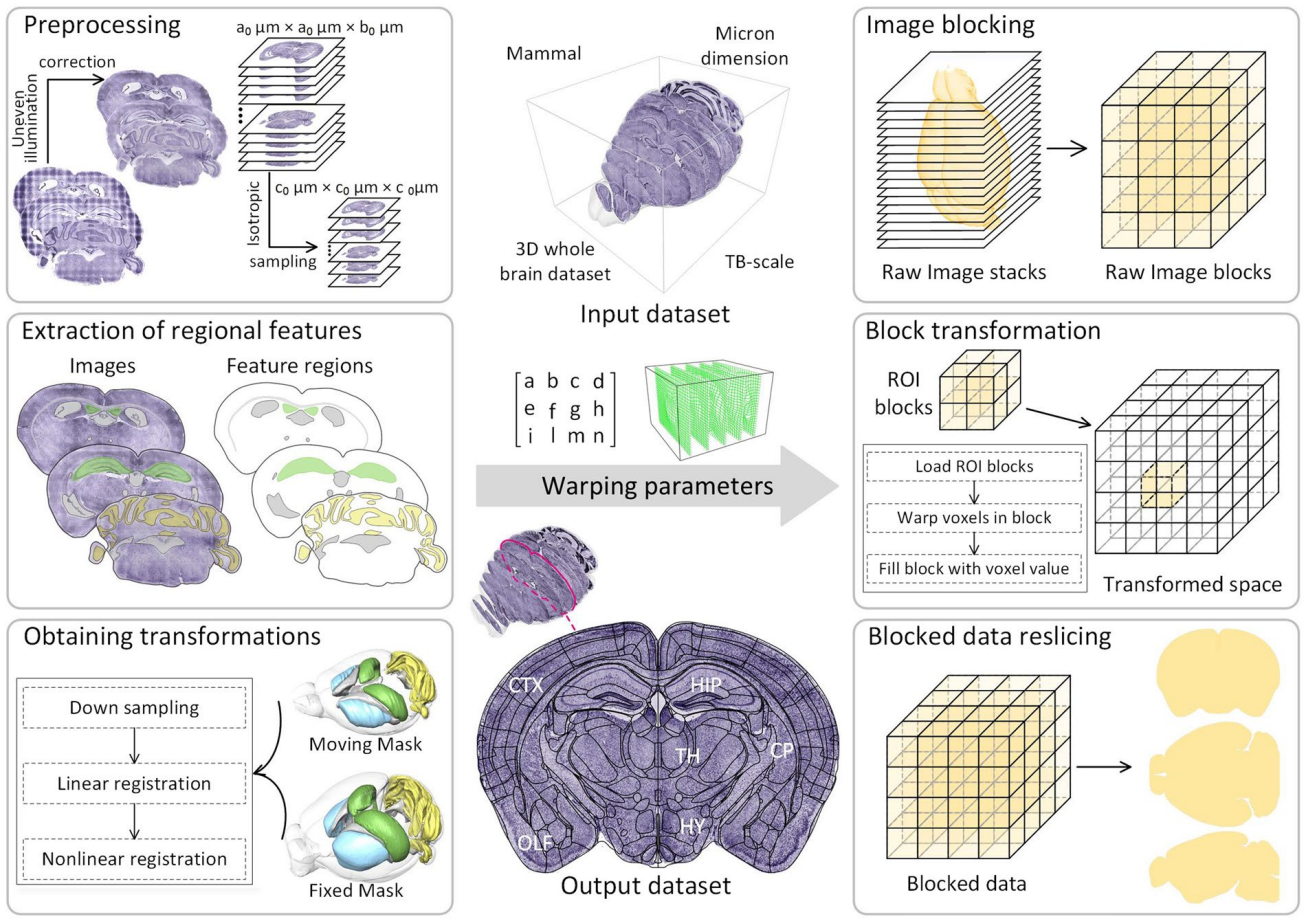
Registration pipeline for large volume brain datasets. Input dataset: mammal, three-dimensional whole brain, micron dimension, TB-scale dataset. The crucial steps to ensure the registration robustness include preprocessing, regional feature extraction, and obtaining transformations, are shown on the left. The obtained warping parameters are input to the right, and the registration process for large-volume brain datasets includes image blocking, block transformation and blocked data reslicing. The registered results are obtained in three anatomical sections. The region lines in ‘Output dataset’ is from the Allen CCF v3, © 2004 Allen Institute for Brain Science. Allen Mouse Brain Atlas. Available from: atlas.brain-map.org.

The complete BrainsMapi pipeline is shown in Fig. 1. We extract anatomically invariant regional features to map brain structures and achieve better registration effects. Compared to observing anatomical points in three-dimensional brain space, extracting the boundaries of specific anatomical regions is more accurate, simpler, and includes information such as shape and size. Based on these findings, we use a parameter obtaining and high-resolution transformation strategy to achieve whole-brain registration of TB-scale datasets at cellular resolution. We describe these two aspects in the following experiments and results.

### Model data

We designed five simple cartoon models with smiling and crying faces (size: 400 × 400 pixel^2^) to demonstrate the effectiveness and robustness of BrainsMapi. The processes are as follows.

(1) Fixed model: A round smiling face in which the eyebrows, eyes, and mouth are marked with different gray level values.

(2) Model 1: We manually designed a deformation, deformed the fixed model, and made it a crying face.

(3) Model 2: To simulate a weak SNR in a real biological dataset, we used the histograms of the corpus callosum (cc), hippocampal region (HIP) and cerebellum (CB) from the Allen CCFv3 Nissl stained dataset and assigned the gray value randomly and automatically to the eyebrows, eyes, and mouth regions of Model 1.

(4) Model 3: We added streak noise to Model 2 using the sin function $y=x\frac{(\sin ((\pi \,/\,w)x)+A-1)}{A}$, where w = 20 and A = 7.

(5) Model 4: Based on Model 3, we set a gray level of zero in a triangular area of the mouth to simulate a torn sample condition; the triangular area was also created manually.

### Biological datasets

We employed 6 whole-brain datasets and 6 sets of metadata to validate BrainsMapi. All the animal experiments followed procedures approved by the Institutional Animal Ethics Committee of Huazhong University of Science and Technology, and this study was specifically approved by this ethics committee. All the methods were carried out in accordance with relevant guidelines and legislation.

Dataset 1^16^ is from the CCFv3 of the Allen Institute, which is an average brain obtained by averaging 1,675 STP brains. The average brain, annotation file, and individual Nissl datasets in four resolution levels (100 μm (132 × 80 × 114 pixel^3^), 50 μm (264 × 160 × 228 pixel^3^), 25 μm (528 × 320 × 456 pixel^3^) and 10 μm (1320 × 800 × 1140 pixel^3^)) are openly available at http://brain-map.org. We adopted the average brain dataset with the highest (10 μm) resolution.

Dataset 2^24^ is a volume of images representing the canonical Waxholm Space (WHS) of the adult C57BL/6 J mouse brain. Five datasets are provided, including T1, T2, and T2* datasets, a Nissl stained dataset, and the manually labeled Atlas dataset. All these datasets are available from https://www.nitrc.org/. Here, we chose the T2*-weight MRI image dataset.

Dataset 3^7^ is a whole-brain image dataset of a Nissl-stained C57BL/6 adult mouse imaged by MOST. The three-dimensional dataset with 1 μm axial resolution and 0.35 μm horizontal resolution was acquired in 7 days using the MOST automatic slicing imaging system. The coronal slice number and the original data size are approximately 11,000 and 4 TB, respectively.

Dataset 4^8^ is from a whole-brain dual-color labeled dataset of the Thy1-GFP M-line of transgenic mice imaged by BPS. We adopted the colocalized fluorescent labeled neurons and counterstained cell bodies dataset, which is brain-wide with a 2 μm axial resolution and a 0.32 μm horizontal resolution. The coronal slice number and original data size of one channel of the dataset are approximately 4,800 and 3 TB, respectively. Here, we adopted the cytoarchitectonic channel dataset.

Dataset 5^8^ is a specifically selected problematic dataset that contains streaks caused by uneven staining and illumination and sample tearing phenomena. This dataset was used for comparison with later results. The imaging system and the staining method are the same as those used to create Dataset 4.

Dataset 6^21^ consists of a collection of published articles from the Division of Neurophysiology, MRC National Institute for Medical Research, London. Briefly, it is a dataset of autofluorescent C57BL/6 mice imaged by STP microscopy. The original dataset has an axial resolution of 5 μm and a coronal resolution of 0.32 μm. The dataset is available from http://www.swc.ucl.ac.uk/aMAP.

Please refer to Table S1 and Fig. S1 for the main characteristics of all the image datasets and metadata.

### Computing environments

In this study, we used two computing devices: a graphical workstation equipped with 20 cores (Intel Xeon E5-2687w × 2) and 128 GB of RAM, and an HPC Cluster with 20 nodes, each equipped with 20 cores (Intel Xeon E5-2660 V3 × 2) and 128 GB of RAM and connected to the Lustre file system via a 10 Gb Ethernet network.

### Image preprocessing

Image preprocessing aims to obtain high quality images for good registration results (Fig. 1 Preprocessing). First, brightness and light-field corrections are used to reduce uneven staining and illumination of optical microscopy images^25^. Then, the corrected dataset is sampled isotopically. Finally, we use the adaptive threshold method^26^ and morphological operations (hole filing, opening and closing operators) to extract the mouse outline and manually fix it.

### Regional feature extractions

The accuracy of selected features is directly related to the registration effects. Here, taking advantage of brain anatomy information, we extracted anatomically invariant regional features to register two brains. The segmented regional features are those with anatomical meanings, that is, brain regions or nuclei. These regional features are conserved; therefore, we can delineate the boundaries of these feature regions in each brain sample.

Here, we selected an interactive segmentation tool, Amira (version 6.1.1; FEI, Mérignac Cedex, France) to perform the feature extraction procedure (Fig. 1: Extraction of regional features). Briefly, the selection of extracted brain regions and nuclei needs to follow these three criteria.

(1) Distributed throughout the brain to ensure the accuracy of brain-wide registration.

(2) Easily identifiable anatomical regions to ensure accuracy and objectiveness for feature extraction.

(3) Registration based on the selection of conserved brain regions or nuclei that are guaranteed to occur in every brain to ensure the registration correctness.

Based on these criteria, we selected the following features from the whole-brain images with the guidance of anatomists: Outline, anterior commissure, olfactory limb (aco)/anterior commissure, temporal limb (act), CB, cc, caudoputamen (CP), fasciculus retroflexus (fr), HIP, medial habenula (MH), facial nerve (VIIn), mammillothalamic tract (mtt), paraventricular hypothalamic nucleus (PVH), pontine gray (PG), lateral ventricle (VL), and fourth ventricle (V4). We also followed the following three criteria to segment these anatomical regions accurately and quickly.

(1) 3D segmentation results: It is necessary to modify the segmentation results repeatedly in the coronal, sagittal and horizontal planes to ensure that the results have continuity in three dimensions.

(2) Accuracy: Segmentation should be conducted under the guidance of anatomical experts and verified by at least three people’s back to back.

(3) Rapidity: We used Amira’s interpolation function to automatically evolve the anatomical regions between two known region images.

We show these regions in Fig. S2. To ensure registration accuracy, the entire regional feature extraction and subsequent transformation obtaining steps were performed at a 10 μm resolution.

### Accurate transformation obtaining

After accurately extracting the features, we need to map them to obtain accurate transformation parameters (Fig. 1: Obtaining transformations). The process of obtaining transformation is an optimization problem. The moving image is warped to the fixed image by the initial transformation parameters, and the similarity metric of the moving and fixed images is used as an energy function. Iteratively, the transformation parameters are updated to achieve an optimal solution and obtain the corresponding transformation. Here, we chose the Symmetric Diffeomorphic Normalization (SyN, ANTs tools, version 2.x)^22^ nonlinear registration method. SyN customizes the symmetry deformation based on the standard method of the Large Deformation Diffeomorphic Metric Matching (LDDMM) proposed by Beg^27^. SyN flexibly records the displacement of each pixel with a large deformation and produces a diffeomorphic transformation of symmetric and invertible displacements (Eq. (1)).

1 $${E}_{syn}(I,J)=\mathop{{\rm{\inf }}}\limits_{\varphi 1}\,\mathop{{\rm{\inf }}}\limits_{\varphi 2}{\int }_{t=0}^{0.5}\{{\Vert {v}_{1}(x,t)\Vert }_{L}^{2}+{\Vert {v}_{2}(x,t)\Vert }_{L}^{2}\}dt+{\int }_{\Omega }{|I({\varphi }_{1}(0.5))-J({\varphi }_{2}(0.5))|}^{2}dt$$

where φ1 and φ2 are the diffeomorphism fields in the opposite directions of domain Ω indexed by time t, t ∈ [0,1], and v_1_ and v_2_ are the opposite-direction velocity fields. Physically, the distance drives the motion of each pixel and is determined based on the image’s potential energy. When the original images are replaced by our extracted regional features, the movement of each pixel is determined by the potential energy of the features, which are not disturbed by the gray signals in the original image. In short, when we replace I and J, which represent the original images with the feature images I_0_ and J_0_, we can obtain accurate transformations.

We used a multiresolution strategy in both linear and nonlinear registrations for acceleration and adopted mutual information as the similarity measure^28^. The entire step to obtain the transformations requires approximately 3 hours. We obtain the linear matrix M, the nonlinear direct displacement field *φ*1 and the inverse field *φ*2.

**Figure 2:**
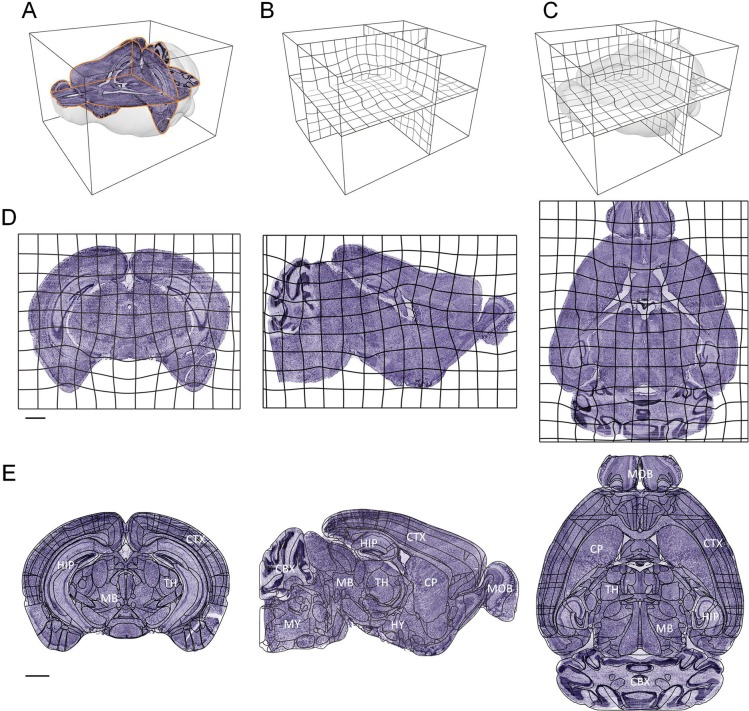
Application of the nonlinear deformation field. (**A**) The three-dimensional rendering of the brain outline and three anatomical sections (coronal, horizontal and sagittal) before nonlinear registration; (**B**) Three-dimensional displacement; (**C**) Three-dimensional deformation field applied to the original three-dimensional dataset; (**D**) A two-dimensional grid shows the application of the deformation fields in the coronal, sagittal, and horizontal planes; (**E**) The registration of coronal, sagittal and horizontal sections corresponding to (**D**), respectively. Scale bars: 1 mm. The region lines in **E** is from the Allen CCF v3, © 2004 Allen Institute for Brain Science. Allen Mouse Brain Atlas. Available from: atlas.brain-map.org.

The displacement is presented in a grid form to intuitively illustrate the nonlinear deformation effect of the diffeomorphism method based on regional features (Fig. 2). Three-dimensional nonlinear displacement (Fig. 2B) and nonlinear registration results (Fig. 2C) are obtained by the nonlinear registration from the linear results (Fig. 2A). The global and local nonlinear deformation effects can be observed from the coronal, sagittal and horizontal sections, respectively (Fig. 2D). For comparison, we also show the registration effects with the atlas line superimposed on the nonlinear results (Fig. 2E).

### Nonlinear transformation for a large volume dataset

In this process, we block large-volume datasets and efficiently transform each block in parallel by performing real-time interpolations of the deformation field instead of upsampling the entire dataset directly, which would lead to TB-scale displacement field generation.

We obtain the transformation parameters after registration at the low resolution (10 μm isotropic) and use them for the high-resolution transformation as follows:

(1) For linear parameters, the low-resolution linear matrix *M* is multiplied by scale matrices to produce a high-resolution linear matrix (*M*′), as shown in Eq. (2);

2 $${\rm{M}}^{\prime} ={{\rm{scaleM}}}_{1}\times M\times {{\rm{scaleM}}}_{2},$$

where *scaleM*_1_ and *scaleM*_2_ are the amplification and shrunken matrices, respectively.

(2) The nonlinear parameters are represented by deformation fields that express the displacement for each voxel in three-dimensional space in a three-channel form (*φ*_*x*_*, φ*_*y*_*, φ*_*z*_). Conversely, we calculate each voxel displacement (*φ*_*x*_ *(x, y, z), φ*_*y*_ *(x, y, z), φ*_*z*_ *(x, y, z)*) by trilinear interpolation (Eq. (3)) during the transformation process according to the low-resolution displacement fields:

3 $$\begin{array}{ccc}\varphi  & = & {\varphi }_{0}(1-{x}_{0})(1-{y}_{0})(1-{z}_{0})+{\varphi }_{1}(1-{x}_{0}){y}_{0}(1-{z}_{0})+{\varphi }_{2}(1-{x}_{0})(1-{y}_{0}){z}_{0}\\  &  & +\,{\varphi }_{3}(1-{x}_{0}){y}_{0}{z}_{0}+{\varphi }_{4}{x}_{0}(1-{y}_{0})(1-{z}_{0})+{\varphi }_{5}{x}_{0}{y}_{0}(1-{z}_{0})\\  &  & +\,{\varphi }_{6}{x}_{0}(1-{y}_{0}){z}_{0}+{\varphi }_{7}{x}_{0}{y}_{0}{z}_{0}\,,\end{array}$$

where *φ*_*1*_, *φ*_*2*_ *…, φ*_8_ are the offset vectors for the eight vertices of a voxel in the low-resolution displacement, *φ* denotes the interpolation result, and (*x*_0_, *y*_0_, *z*_0_) are the relative coordinates in the voxel box.

(3) P(x, y, z) denotes the space coordinate of voxel P before registration in the high resolution, and the coordinates after linear and nonlinear registration are P’ (x’, y’, z’) and P”(x”, y”, z”), respectively. Here, *s* indicates a scaling factor, which is the quotient of the low-resolution and high-resolution ratios. Then, the mapping relationship is shown in Eq. (4):

4 $$\begin{array}{c}[{\rm{x}}^{\prime} ,{\rm{y}}^{\prime} ,{\rm{z}}^{\prime} ]=[{\rm{x}},{\rm{y}},{\rm{z}}]\times {\rm{M}}^{\prime} \\ \{\begin{array}{c}{\rm{x}}^{\prime\prime} ={\rm{x}}^{\prime} +{\rm{s}}\times {{\rm{\varphi }}}_{{\rm{x}}}({\rm{x}}/{\rm{s}},{\rm{y}}/{\rm{s}},{\rm{z}}/{\rm{s}})\\ {\rm{y}}^{\prime\prime} ={\rm{y}}^{\prime} +{\rm{s}}\times {{\rm{\varphi }}}_{{\rm{y}}}({\rm{x}}/{\rm{s}},{\rm{y}}/{\rm{s}},{\rm{z}}/{\rm{s}})\\ {\rm{z}}^{\prime\prime} ={\rm{z}}^{\prime} +{\rm{s}}\times {{\rm{\varphi }}}_{{\rm{z}}}({\rm{x}}/{\rm{s}},{\rm{y}}/{\rm{s}},{\rm{z}}/{\rm{s}})\end{array}\end{array}$$

After obtaining the mapping relationship, we use TDat tools^29^ to partition the raw image sequences into cubic blocks (Fig. 1: Image blocking). The transformed space is precalculated based on the sizes of the fixed images and the scaling factor (*s*). Next, we apply transformation parameters for each block in transformed space separately. The details are as follows (Fig. 1: Block transformation).

(1) For each block in transformed space, we calculate the block involved in the original space by transforming all the points on the six surfaces of the block using the mapping relationship (Eq. (4)). Then, the corresponding blocks (ROI blocks) in the original space are loaded into memory. Generally, a transformed block only needs to import about 4~20 corresponding blocks in original space.

(2) For every voxel in a block, we calculate the spatial coordinate of the corresponding voxel in the ROI blocks using the mapping relationship (Eq. (4)) and use the grayscale value of the coordinate for this voxel.

(3) Each block is written to a disk in three-dimensional image format.

After completing the above process, we acquire the registration of the three-dimensional image dataset at the original resolution. Finally, the registered two-dimensional image sequences of the three anatomical sections (coronal, horizontal, sagittal) are generated by reslicing (Fig. 1: Blocked data reslicing).

We used the following ideas to optimize the calculation efficiency. First, parallel implementations of the process-level message passing interface (MPI) and thread-level OpenMP are applied to the data reading, writing and calculating operations in a multicomputer environment. Second, we use video coding^30^ to greatly compress the image datasets, reducing the burden of a large number of I/O operations during transformation. Third, image data external to the brain are considered as redundant to reduce the number of calculations.

### Vectorized dataset registration

Integrating datasets such as cells^31,32^, neurons^8,33^, and vessels^34^ requires mapping them into a standard space. These vectorized datasets are constructed of point sets and coordinates. Using neurons as an example, traced neurons are saved as SWC files in the form of sequence points. As an incidental outcome of large-volume transformation, we can easily register these vectorized datasets.

### Multilevel quantitative evaluation

Here, with reference to the aMAP^21^ evaluation method, we designed a set of multilevel quantitative assessment methods, from coarse to fine, and assessed the registration results from the brain-region level to the identifiable nuclei level at a 10 μm resolution objectively.

By design, we first assess the accuracy at the brain-region level, a coarse assessment. A total of ten brain regions are chosen throughout the brain: Outline, CB, CP, hindbrain (HB), HIP, hypothalamus (HY), isocortex (ISO), midbrain (MB), pons (P) and thalamus (TH). We manually segment the moving image after registration, but regard the segmentation results as a silver standard^11^ instead of a golden standard. To reduce the workload, we do not segment each complete brain region slice by slice; instead, we selected 50 images at the same interval in each region and segmented those slice by slice. All the manual segmentation results are referenced to the Allen CCFv3. The Dice score^35^ (Eq. (5)) was selected as the evaluation measure:

5 $${\rm{Dice}}\,{\rm{score}}=\frac{2\times |{\rm{I}}\cap {\rm{J}}|}{|{\rm{I}}|+|{\rm{J}}|},$$

where *I* is the moving image after registration, *J* is the fixed image, and ∩ denotes the intersection of two images. The Dice score is calculated in two-dimensions, and the number of Dice scores for each brain region is 50.

To design an identifiable nuclei level evaluation at a 10 μm resolution, we selected nine nuclei selected brain-wide (anterior cingulate area (ACA), primary visual area (VISp), primary somatosensory area (SSP), reticular nucleus of the thalamus (RT), ventromedial hypothalamic nucleus (VMH), periaqueductal gray (PAG), subiculum (SUB), entorhinal area, lateral part (ENT1), medial vestibular nucleus (MV)). Twenty-two trained technicians segmented these regions into five registered datasets (four types). First, we select a representative coronal section in the CCFv3 for each nucleus; a stack (40 sequences) of corresponding positions in the registered datasets is also provided. Then, these 22 individuals were tasked with identifying a single coronal section from the stack that they considered the most similar to the reference coronal section and segmenting it. Based on this procedure, the STAPLE algorithm^36^ was used to fuse the 22 human segmentation results and obtain the STAPLE results. The Dice scores of each segmentation result and the STAPLE results are considered as the human performance (HP), while the Dice scores of the CCFv3 and STAPLE results are calculated as the registration performance (RP). Then, a correlation analysis was performed.

## Experiments and Results

### A model experiment for robust demonstration

To demonstrate the effectiveness of our designed regional features, we customized a model experiment to compare BrainsMapi with other registration methods under different situations (see Materials and Methods).

**Figure 3:**
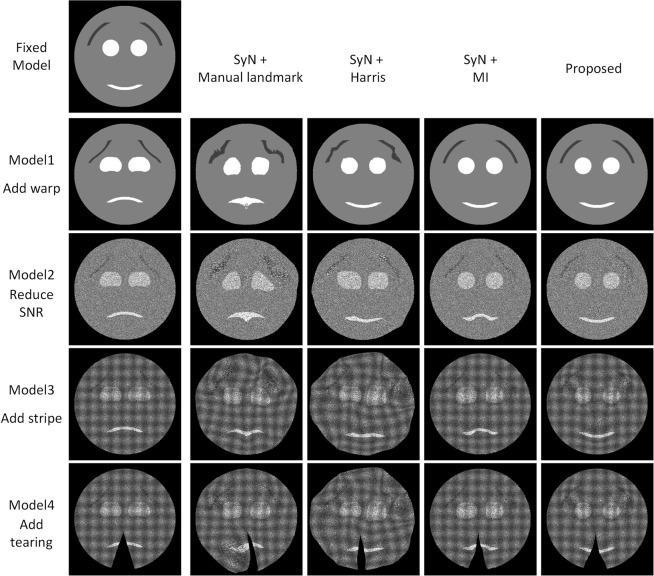
Registration effects on model data. Comparison of different registration methods. The first row of smiles is the fixed model. Rows 2 to 5 correspond to the registration results of the manual and automatic point-based registration methods, the gray-level-based registration method and the proposed BrainsMapi in different modeled situations, respectively.

A crying face turning into a smiling face demonstrates that good registration effects have been achieved. We manually segmented several regions (e.g., mouth, eyes) in synthetic images and registered them to the fixed model. The last column in Fig. 3 shows that regardless of the deformation, weak SNR or streak noise, BrainsMapi can obtain good registration results. Although the tearing effects in images are not fixed after registration, BrainsMapi prevents incorrect registration results.

In contrast, we demonstrate the effects of other registration strategies on the model data, mainly focusing on the points- and gray-level-based methods. The results of manually selecting feature points (Symmetric Diffeomorphic Normalization (SyN) from 40–50 manually selected points) involve some randomness due to the limited numbers and inconsistent locations of the selected feature points (Fig. 3, second column). It is difficult to extract accurate feature points automatically (SyN with Harris) from images with weak SNR and strong noise, which leads to inaccurate registration (Fig. 3, third column). The gray-level-based registration method (SyN with mutual information (MI)) is also difficult to process under weak SNR, strong noise interference and torn images (Fig. 3, fourth column). Additional three-dimensional model demonstrations are provided in the Supplementary Materials (Fig. S3).

We also compared and quantified the registration results on the real biological dataset (Fig. S4). We selected 50 pairs of feature points from the whole brain (Fig. S4D), but the results are still poor (Fig. S4A). Moreover, we found it difficult to select points accurately (especially in the z direction) and determine which slice corresponds to which point. We emphasize that anatomical regional features can better ensure the accuracy and robustness of registration.

### Registering sample tearing and streak images for intramodal registration

The complications simulated in the model data also exist in brain images. There are differences in image quality even with images in the same modality. These images are easily disturbed during sample preparation and imaging processes and can include obvious streaks caused by uneven illumination (Fig. S5). Nevertheless, the image signals of the intramodal datasets are similar after the preprocessing step; in particular, they have small individual differences, and hence, their registration is relatively easier.

**Figure 4:**
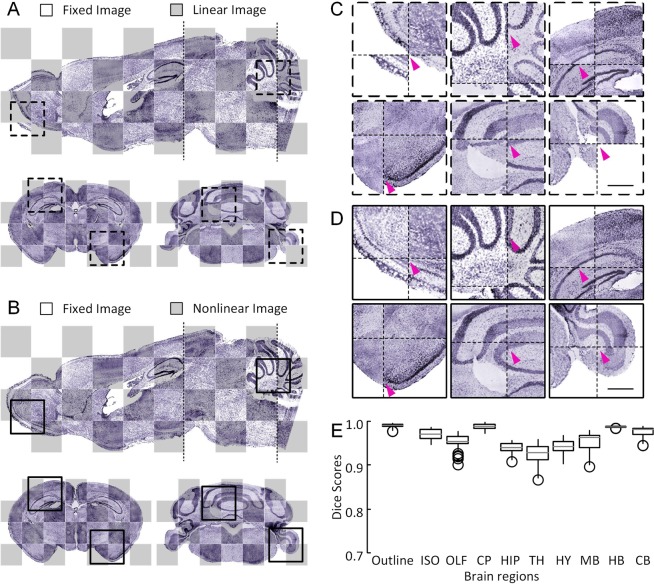
Registration effects for sample torn and streaked images. (**A**) The checkboard reconstructions show the linear registration of one sagittal and two coronal slices as indicated by dashed lines in the sagittal slice. The projection thickness is 10 μm. (**B**) The checkboard reconstructions show the nonlinear registration results of one sagittal and two coronal slices as indicated by the dotted lines in the sagittal slice. The projection thickness is 10 μm. (**C**) The enlarged views of the linear results of local regions indicated by the corresponding dotted boxes in (**A**). (**D**) The enlarged views of the nonlinear results of local regions, indicated with corresponding dotted boxes in (**B**). The thin cross-dotted lines in (**C**,**D**) represent the checkboard grid. (**E**) A quantitative evaluation at the brain-region level. The small purple arrows indicate misalignment positions that are corrected after nonlinear registration.

Using Dataset 4 as a reference brain, we present an intramodal registration by aligning Dataset 5 to the reference brain based on BrainsMapi. We visualize the registration results in the form of checkerboards in Fig. 4. The global orientation of the mouse brain is corrected after the linear registration (Fig. 4A), while the local brain regions and nuclei are adjusted after the nonlinear registration (Fig. 4B). Compared to the linear results (Fig. 4C), the nonlinear registration resulted in good local correction of regions such as the olfactory areas (OLF), CB, HIP, and the paraflocculus (PFL) (Fig. 4D). The small purple arrows in Fig. 4C,D indicate that misalignment positions that are corrected after the nonlinear registration.

In addition, we evaluated the registration accuracy at the brain-region level (see the Materials and Methods section). In the box plot (Fig. 4E), the median Dice score for all brain regions is between 0.9 and 0.99. Generally, a Dice score above 0.8 indicates that a good registration effect has been achieved^11,22^.

### Register individuals with large differences and deformations for intermodal registration

**Figure 5:**
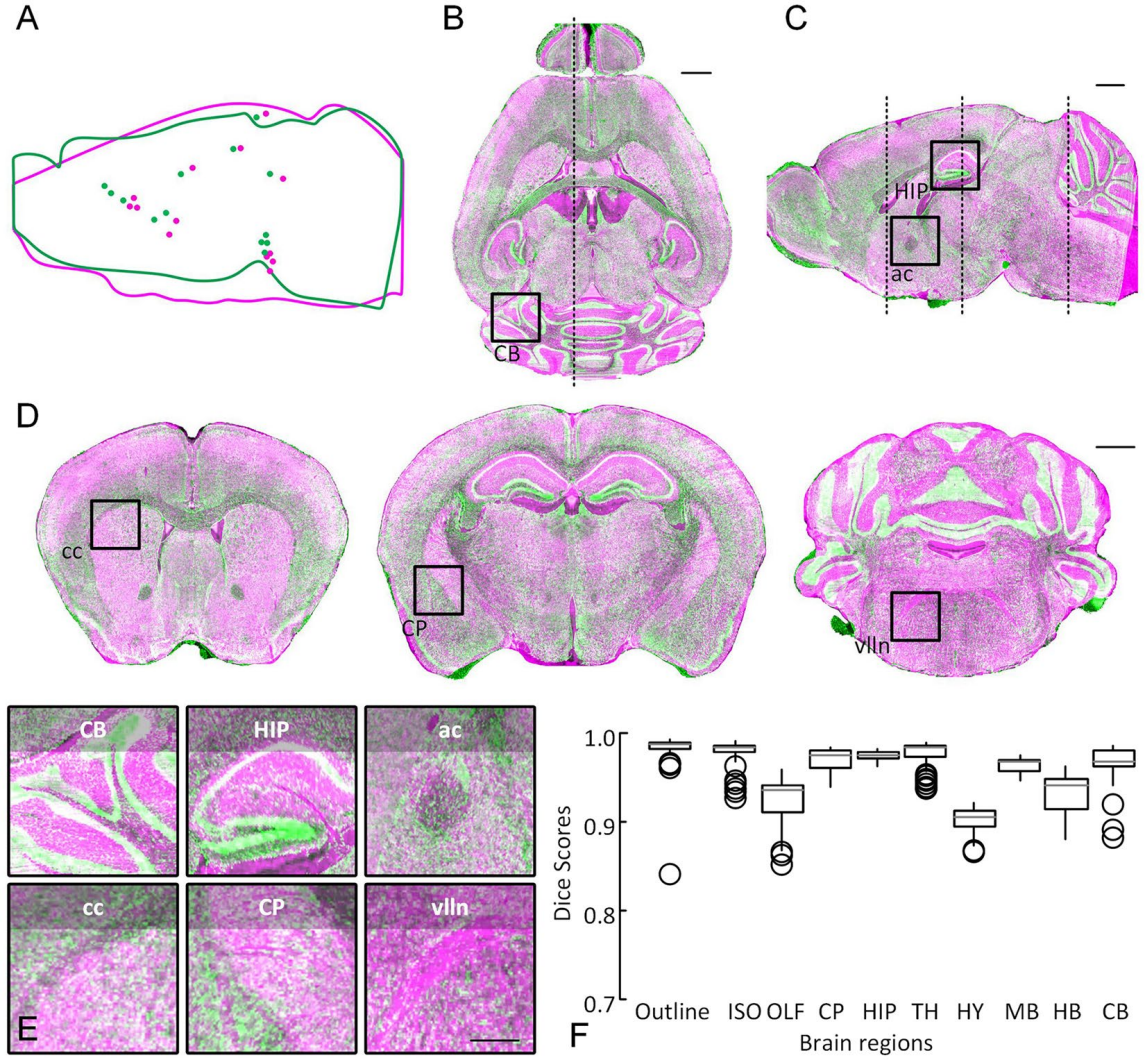
Registration effects for large different individuals. (**A**) A schematic diagram of thirteen pairs of landmarks and brain outlines. The fixed image is shown in purple, and the moving image is shown in green. (**B**) A horizontal reconstruction at the location indicated by a dot outline in (**A**) merged the fixed image (purple) with the registered moving image (green). The projection thickness is 10 μm. (**C**) A sagittal reconstruction at the location indicated by a dotted line in (**B**) merged the fixed image (purple) with the registered moving image (green). The projection thickness is 10 μm. (**D**) The 10 μm thick projection of several merged coronal sections indicated by the dotted line in (**C**). (**E**) Merged image of the enlarged views of local regions indicated by the text annotations and corresponding boxes in **(B**–**D)**. (**F**) A quantitative evaluation at the brain-region level. Scale bars: (**B**–**D**) 1 mm, (**E**) 0.5 mm. cerebellum (CB), hippocampal region (HIP), anterior commissure, olfactory limb (aco), corpus callosum (cc), caudoputamen (CP), facial nerve (VIIn). Waxholm Space data provided courtesy of G. Allan Johnson, Ph.D., Duke Center for *In Vivo* Microscopy.

In addition to image quality, there are general differences among individuals and deformations, especially the deformations incurred during the brain sample preparation stage of mesoscopic optical imaging. The registration of these large deformation datasets is more difficult. Here, we expect to show registration results of individuals with large differences and deformations. By applying a method for measuring the distance between anatomical landmarks^37^, we found that the greatest difference in individuals and deformation pairs was in Datasets 2 and 4 among the following four datasets (Dataset 2 (MRI), Dataset 3 (MOST), Dataset 4 (BPS) and Dataset 6 (STP)) (Fig. S6). We also show the distance between these two datasets in Fig. 5A (median 596.9 μm).

Dataset 4 is registered using Dataset 2 as a reference brain. By merging Dataset 4 (green channel) with Dataset 2 (purple channel), we present the intermodal registration results for horizontal (Fig. 5B), sagittal (Fig. 5C) and several coronal sections (Fig. 5D). The brain outline and large brain regions are well aligned. Moreover, enlarged views are provided in Fig. 5E. Nuclei such as CB, HIP, aco, cc, CP, and VIIn can also be well mapped.

We also conducted a quantitative assessment of brain-region levels. In the box plot (Fig. 5F), the median Dice score for all the brain regions is between 0.9 and 0.99.

### Register to a reference atlas for multimodal registration

We present a special registration type in this section that is registered to the reference atlas. Using direct or inverse registration to the reference brain space, we are able to acquire the spatial information of the original dataset, which is the key to integrating multi-modal image datasets into a common brain space.

**Figure 6:**
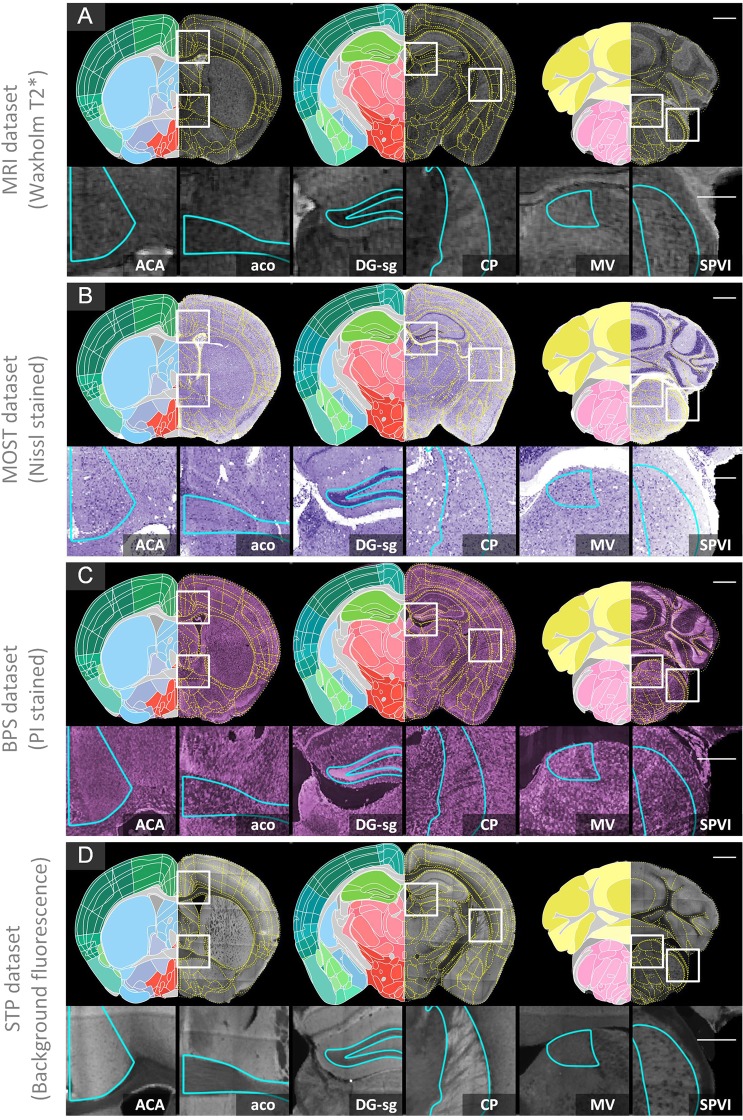
Registration effects for multimodal datasets. The 10-μm thick projections of three coronal sections: the left half is the Allen CCFv3, and the right half is the yellow dotted line of the Allen CCFv3 superimposed on the original image. The enlarged views of several local regions provided below are indicated by the white boxes in the above coronal sections. (**A**) Dataset 2 (MRI), (**B**) Dataset 3 (MOST), **(C**) Dataset 4 (BPS), (**D**) Dataset 6 (STP). Scale bars: coronal 1 mm, detail 0.5 mm. Anterior cingulate area (ACA), anterior commissure, olfactory limb (aco), dentate gyrus, granule cell layer (DG-sg), caudoputamen (CP), medial vestibular nucleus (MV), spinal nucleus of the trigeminal, interpolar part (SPVI). Allen CCF v3, © 2004 Allen Institute for Brain Science. Allen Mouse Brain Atlas. Available from: atlas.brain-map.org. Waxholm Space data provided courtesy of G. Allan Johnson, Ph.D., Duke Center for *In Vivo* Microscopy. STP dataset provided courtesy of Troy Margrie, UCL Professor of Systems Neuroscience.

We register four types of datasets (Datasets 2–4 and 6) to Allen CCFv3 (Dataset 1) and present the registration results. From top to bottom (Fig. 6), the images correspond to the registration results of Datasets 2–4 and 6. We selected three coronal sections with the form of the Allen CCFv3 on the left; the yellow dotted line shows the Allen CCFv3 superimposed on the original image in the results. The brain outline and large brain regions, such as HIP, TH, HY, MB and CB, have good alignment with the reference atlas as judged from their spatial orientations and anatomical boundaries. Moreover, we present enlarged views of local regions of nuclei for each data type. The nuclei with distinct boundaries, such as act, dentate gyrus, granule cell layer (DG-sg), and CP, are well aligned. For ACA, the upper and lower boundaries can be judged by the original image. For MV and the spinal nucleus of the trigeminal, interpolar part (SPVI) with inconspicuous boundaries, the accuracy can be judged by the brain spatial orientation. All these areas are well aligned.

**Figure 7:**
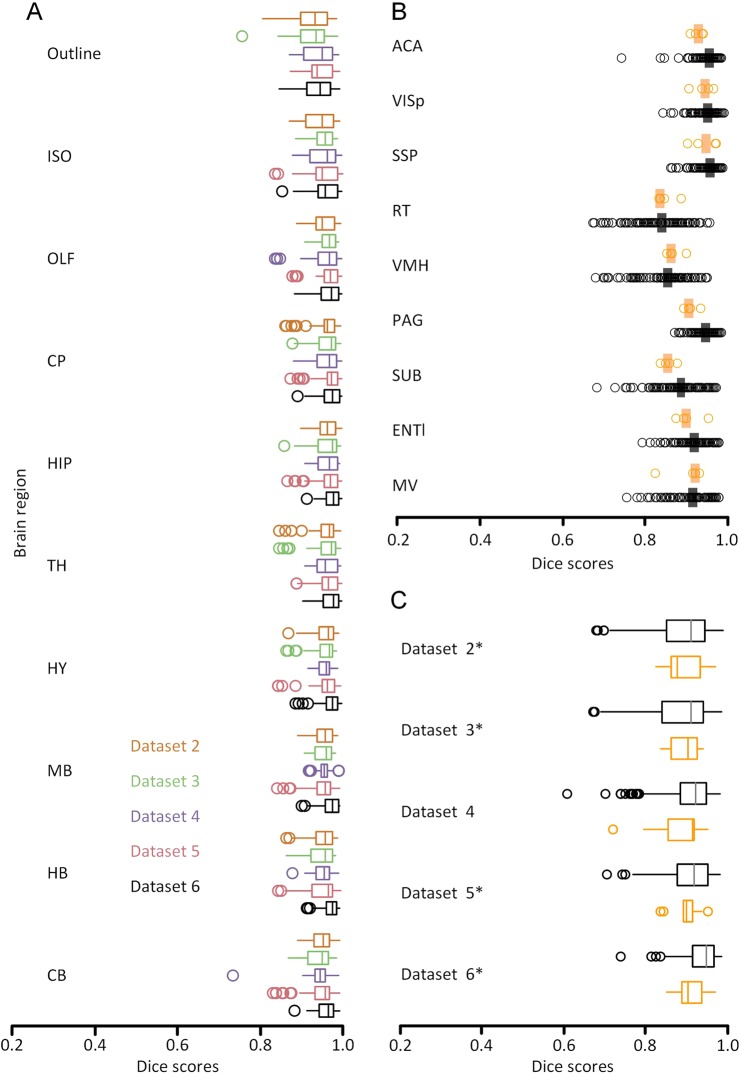
Multilevel quantitative assessment results. (**A**) The assessment at the brain-region level: boxplot of Dice scores for ten brain regions of five brain datasets (indicated with different colors) (n = 5). (**B**) The assessment at the nuclei level: Dice scores of registration performance (RP, orange) and human performance (HP, black) are grouped by structure (n = 4). The vertical lines indicate the median scores. (**C**) The assessment at the nuclei level: Dice scores of registration performance (RP, orange) and human performance (HP, black) are grouped by dataset (n = 5). The brains used in (**B**) are marked with an asterisk.

According to the above registration results, we first assess the accuracy at the brain-region level to form a rough assessment (See Materials and Methods). We evaluated ten brain regions in the brain-wide data of the five datasets. The results are shown in box plots (Fig. 7A). The median Dice scores of all the brain regions and datasets are above 0.9.

In addition to the assessment of identifiable nuclei level at a 10 μm resolution, nine brain anatomical structures are selected (see the Materials and Methods section). When we grouped the results by HP or RP, the medians acquired by HP (Fig. 7B, black) were not significantly different from those acquired by RP (Fig. 7B, orange) (Mann-Whitney U-test, score of 0.90 versus 0.92, P = 0.054; n = 4 brains, 9 structures, 22 human raters). When we grouped these scores by nucleus, there were no significant differences between HP and RP in eight structures except that the PAG in HP is significantly better than the RP at segmenting the PAG regions (Fig. 7B) (Mann-Whitney U-test, score of 0.94 versus 0.90, P = 0.017; n = 4 brains, 9 structures, 22 human raters). When we grouped the results by datasets, there was no significant difference between HP and RP for the four individual brains (Fig. 7B) (Mann-Whitney U-test, P > 0.06).

All these results demonstrate that the method proposed in this paper has extremely high accuracy at the brain-region level and its results do not differ significantly from manual results performed at the nuclei level at a resolution of 10 μm.

### Whole-brain registration for TB-scale dataset at single-cell resolution

We used the low-resolution registration results (10 μm) to demonstrate the robustness and accuracy of BrainsMapi in the previous section. Additionally, we achieved registration of the TB-scale dataset at single-cell resolution.

Nonlinear registration for a large-volume dataset is able to correct nonuniform deformations in brains and integrate datasets at the cellular level. The original images can be registered to the standard brain space without any loss of resolution, while in the past, they were analyzed under original unregistered images. We can now maintain the original resolution while directly obtaining the analyzed results from the registered high-resolution images.

**Figure 8:**
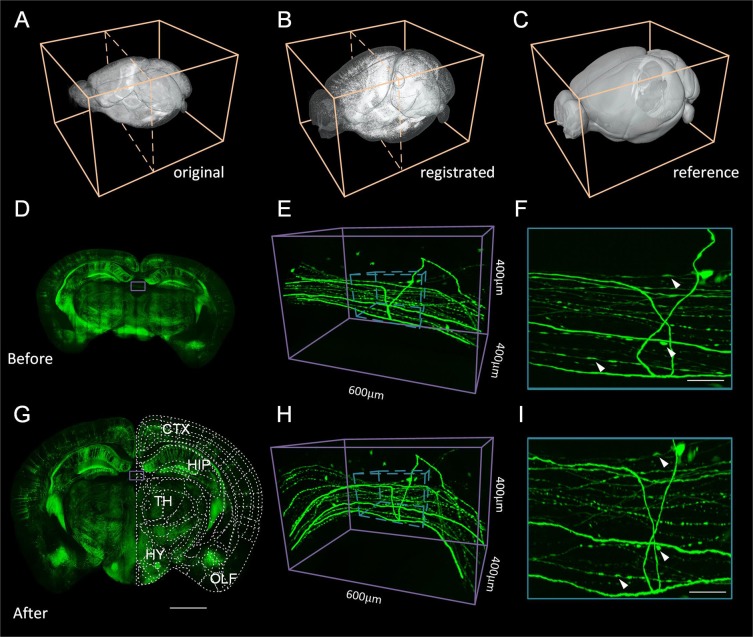
Registration effects for the whole-brain at single-cell resolution. (**A**) The three-dimensional rendering of the original GFP channel of the Thy1-GFP M-line of transgenic mice; (**B**) The three-dimensional rendering of the registered GFP channel; (**C**) The three-dimensional rendering of the brain outline of the Allen CCFv3; (**D**) The 256 μm thick coronal projection of the original image; (**E**) The enlarged three-dimensional rending view at the location indicated by the blue box in (**D**,**F**) The enlarged 256 μm thick projection view at the location indicated by the blue cuboid in (**E**,**G**) The 256 μm thick coronal projection of the registered image; (**H**) The enlarged three-dimensional rendered view at the location indicated by the blue box in (**G**,**I**) The enlarged 256 μm thick projection view at the location indicated by the blue cuboid in (**H**). The small arrows indicate the corresponding fine structures before and after registration. Allen CCF v3, © 2004 Allen Institute for Brain Science. Allen Mouse Brain Atlas. Available from: atlas.brain-map.org.

Dataset 5, which has a 0.32 μm × 0.32 μm × 1 μm original resolution, is sampled to 0.32 μm isotropic. The dataset is approximately 20 TB in size. The processed images are aligned to the Allen CCFv3 in three dimensions (Fig. 8A–C), and the brain regions are matched accurately (Fig. 8G). We also present the difference before and after registration of coronal images (Fig. 8D,G). For the finer-scale enlarged views, the neuron fibers are simultaneously corrected and maintain the continuous structure (Fig. 8E,H). The fine and weak signals also remain consistent after registration. We also demonstrate the high-resolution registration results on the Nissl stained dataset (Fig. S7).

**Figure 9:**
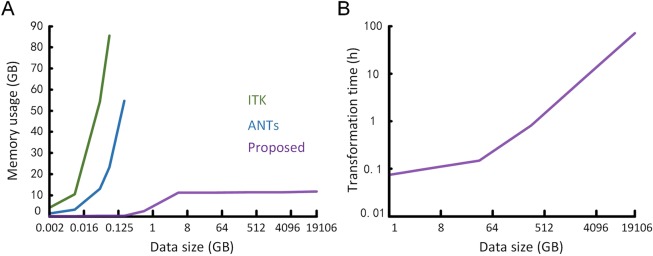
Performance of registration for large-volume datasets. (**A**) Comparisons of the memory consumptions for different dataset sizes between our method and two existing registration tools (ITK and ANTs). (**B**) The changes in the time taken for transformations as the data size increases. The datasets are sampled to multiresolution for comparisons; their sizes ranged from 0.001 GB to 19,106 GB.

Finally, we evaluated the registration performance on a large-volume dataset (Fig. 9 and Table S2). As the data size increases, BrainsMapi memory consumption remains stable at approximately 14 GB, while the memory consumptions of ITK and ANTs increase sharply (Fig. 9A). Furthermore, BrainsMapi can process a 1 TB dataset within several hours and requires only 24 hours for 20 TB datasets (Fig. 9B).

### Registering existing metadata to a reference atlas

Metadata refers to digitized, vectorized information acquired from the original image dataset. These metadata are scattered across different brain spaces, individual laboratories and projects. As a part of the BrainsMapi features, a vectorized dataset can be registered into a standard brain space to complete the integration and localization.

**Figure 10:**
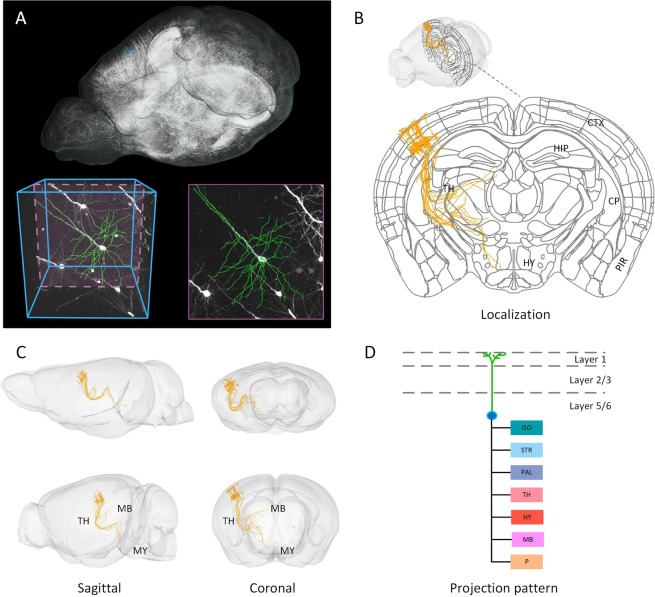
Registration effects for existing metadata. (**A**) The three-dimensional rendering of the GFP channel of Thy1-GFP M-line transgenic mice, three-dimensional and two-dimensional vectorized neurons (Metadata 1); (**B**) The coronal projection of the Allen CCFv3 with vectorized neurons after registration indicated in the three-dimensional rendering in (**B**,**C**) The three-dimensional rendering of three anatomical projections (sagittal and coronal) of the outline and neurons before (**C**, top) and after registration (**C**, bottom); (**D**) The projection pattern of a single neuron. Allen CCF v3, © 2004 Allen Institute for Brain Science. Allen Mouse Brain Atlas. Available from: atlas.brain-map.org.

By aligning metadata to the Allen CCFv3, we present the registration results of vectorized neurons. The vertebral neurons are manually traced from unregistered Brain 4 (Fig. 10A). We deform these metadata and automatically obtain the projection patterns from the cortex (CTX) through the TH and then project them to the MB and medulla (MY) (Fig. 10B). For comparison, we also present the results before and after registration in the sagittal and coronal (Fig. 10C) planes. The projection pattern of each neuron can be automatically calculated by the localization results as well (Fig. 10D).

Additionally, we present the registration results of some other metadata, including segmented blood vessels (Fig. S8A), traced neurons (Fig. S8E), and counted cell bodies (Fig. S8I). Automatic location and integration of these scattered metadata from different sources can be completed by BrainsMapi.

## Discussion

In this paper, we propose a robust registration interface for large-volume brain datasets, named BrainsMapi. We use anatomically invariant regions during the registration to ensure the accurate extraction of a large number of features, enabling BrainsMapi to register various datasets accurately. Furthermore, by obtaining both the low-resolution parameters and conducting a high-resolution transformation strategy, we can realize registrations of TB-scale whole-brain datasets by block warping. We demonstrate the robustness of our registration method on both model data and real brain images and present the nonlinear registration results of a three-dimensional whole-brain fine image dataset at single-neuron resolution. Additionally, the labeled and existing vectorized datasets are registered to a standard brain space. Finally, we design an objective multilevel evaluation method to prove the accuracy of our approach.

The regional features of our method are not limited to cytoarchitectural image data, reflecting its wide applicability. As long as the anatomical region can be identified in the image, the method can be used for registration. Moreover, the selection of regional features is not absolute and can be autonomous based on the image characteristics or experimental requirements until the registration results meet expectations. An ideal situation is to select all the brain regions for registration; this approach obtains the best results, but the cost is high. Therefore, we analyzed the relationship between the selected features and BrainsMapi’s performance (Fig. S9) and found that with the continuous addition of features, the accuracy of local regions related to the features is improved. Additionally, due to the different registration tasks and different user skills, it is difficult to quantitatively evaluate the time cost of extracting features: for some simple and small features, such as MH, fr and mtt, feature extraction takes only a few minutes; for more complex and large features, such as the outline and CB, it takes between half an hour and an hour approximately. In this paper, we recommended 14 regions based on experience to obtain sufficiently accurate results at lower cost. A skilled person may require only about 4 to 6 hours to perform the feature extraction steps needed to obtain accurate registration results.

Another important aspect of this paper is the registration method for large-volume datasets. It is difficult to achieve whole-brain registration with accurate positioning at the cellular level due to hardware limitations and the complexity of the registration algorithms, and the highest 3D mouse brain atlas in the world has only a 10 μm resolution (CCFv3). Therefore, BrainMapi’s high-resolution registration strategy is currently the most appropriate. Certainly, once a 3D brain atlas at the cellular level is available, accurate registration to the mesoscopic level will be our ultimate goal. In addition, our method is highly scalable and is very promising for future applications to petavoxel datasets, such as the marmoset and human brain datasets.

Neuroscientific analysis with brain spatial orientation requires matching the dataset to a standard brain space coordinate system to obtain anatomical boundaries. More general and common analysis skills involve combining multimodality and multiscale datasets, such as MRI, optical imaging, or even electron microscopy datasets, to reveal the structural and functional relationships in the brain,. The integration of mesoscopic and macroscopic datasets is more meaningful, valuable and efficient. Many projects^38–40^ have used registration techniques to integrate various datasets—even the recent international brain projects^1^ desire to develop a powerful, standardized and industrialized framework to integrate multiscale, multimode and massive datasets for studies on brain function mapping, disease models, and behavioral cognition^41^. BrainsMapi is highly compatible with the data integration requirements. It can accurately register various image datasets and existing vectorized metadata and handle high-throughput, TB-scale, large-volume whole-brain datasets, providing a complete and effective pipeline for brain data integration.

The datasets and codes of this study are available from the corresponding author on reasonable request.

## Supplementary information

Supplementary Information.
