# Supplementary material for: A Robust Image Registration Interface for Large Volume Brain Atlas
Source: Sci Rep. 2020 Feb 7;10:2139. doi: 10.1038/s41598-020-59042-y (PMC7005806; doi:10.1038/s41598-020-59042-y)
Supplement: Supplementary file 1 — Supplementary Information. [file 41598_2020_59042_MOESM1_ESM.pdf]

Supplementary materials

A Robust Image Registration Interface for Large Volume Brain Atlas

Hong Ni[1,2,+], Chaozhen Tan[1,2,+], Zhao Feng[1,2], Shangbin Chen[1,2], Zoutao Zhang[1,2], Wenwei Li[1,2], Yue Guan[1,2], Hui Gong[1,2,3], Qingming Luo[1,2], Anan Li[1,2,3,*]

[1] *Britton Chance Center for Biomedical Photonics, Wuhan National Laboratory for Optoelectronics-Huazhong University of Science and Technology, Wuhan 430074, China*

[2] *MoE Key Laboratory for Biomedical Photonics, School of Engineering Sciences, Huazhong University of Science and Technology, Wuhan 430074, China*

[3] *HUST-Suzhou Institute for Brainsmatics, Suzhou 215000, China.*

[+] Equal contribution.

[*] Corresponding author.

E-mail: aali@mail.hust.edu.cn (Li A)

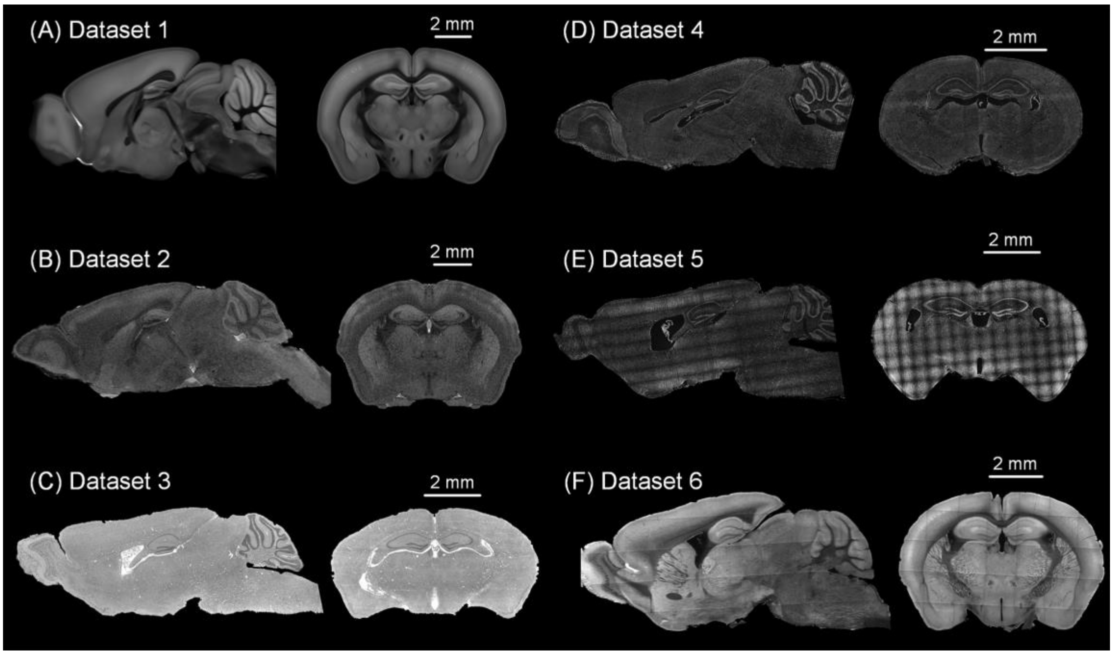

Supplementary Figure 1 The display of the image datasets

The reconstructed sagittal and coronal planes of six sets of image datasets. **A.** Dataset 1 is from the Allen CCFv3. **B.** Dataset 2 is the T2*-weight MRI Waxholm image dataset from NITRC. **C.** Dataset 3 is the whole-brain image dataset of Nissl-stained C57BL/6 adult male imaged by MOST. **D.** Dataset 4 is from the whole-brain dataset of dual-color labeled Thy1-GFP M-line transgenic mice imaged by BPS. **E.** Dataset 5 is from the whole-brain dataset of dual-color labeled Thy1-GFP M-line transgenic mice imaged by BPS, which is specifically selected problematic for comparative analysis. **F.** Dataset 6 is an autofluorescent C57BL/6 male imaged by STP microscopy. Scale bars: 2 mm. Allen CCF v3, © 2004 Allen Institute for Brain Science. Allen Mouse Brain Atlas. Available from: atlas.brain-map.org. Waxholm Space data provided courtesy of G Allan Johnson, Ph.D., Duke Center for In Vivo Microscopy. STP dataset provided courtesy of Troy Margrie, UCL Professor of Systems Neuroscience.

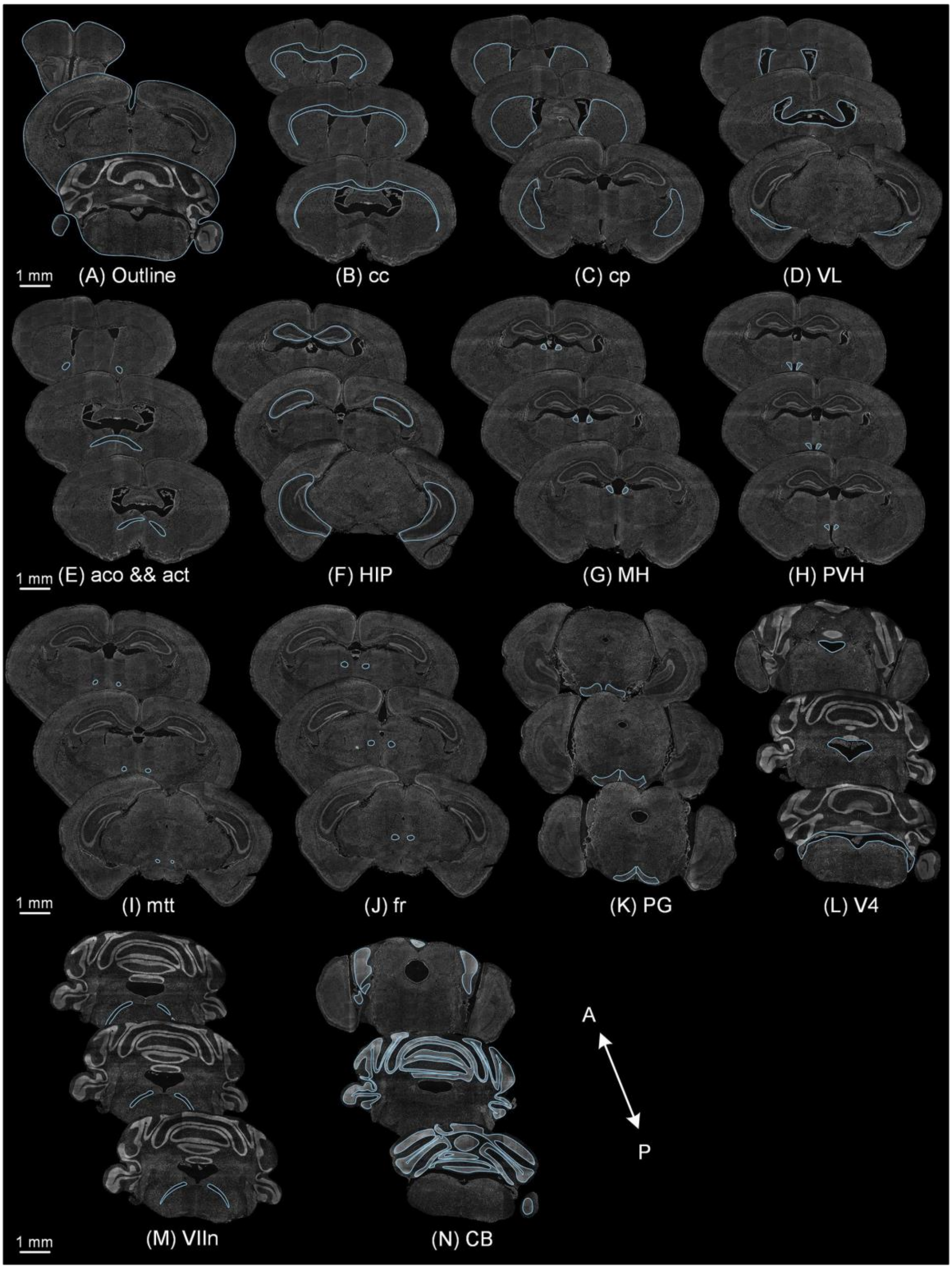

Supplementary Figure 2 The effects of 14 extracted brain regions

The effects of 14 extracted feature regions by using the form of feature lines superimposed on coronal images. **A.** Outline, **B.** cc, **C.** cp, **D.** VL, **E.** aco/act, **F.** HIP, **G.** MH, **H.** PVH, **I.** mtt, **J.** fr, **K.** PG, **L.** V4, **M.** VIIn, **N.** CB.

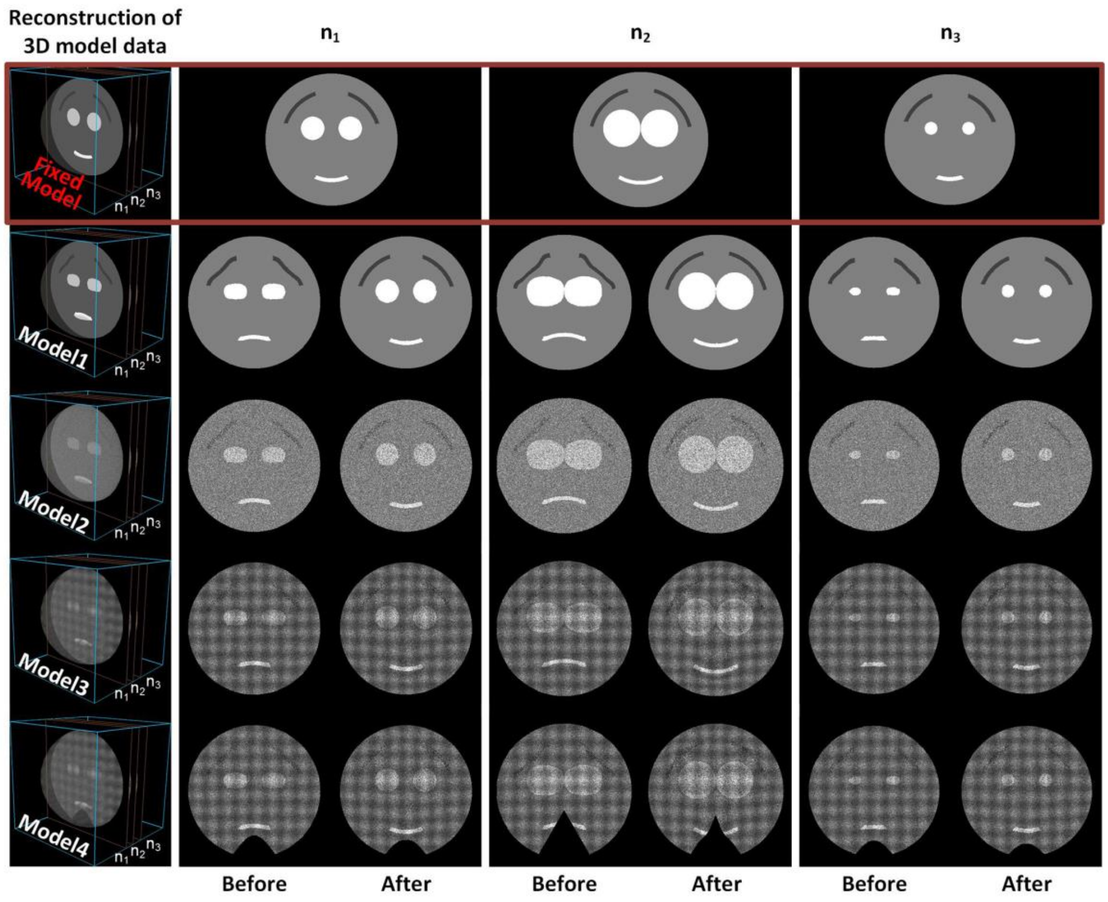

Supplementary Figure 3 The registration effects of three-dimensional model data

The first row of smile is the fixed model, and n_1, n_2 and n_3 represent three 2D slices. Row 2-5 are the registration results of model 1-4, and n_1, n_2 and n_3 represent three 2D slices before and after registration.

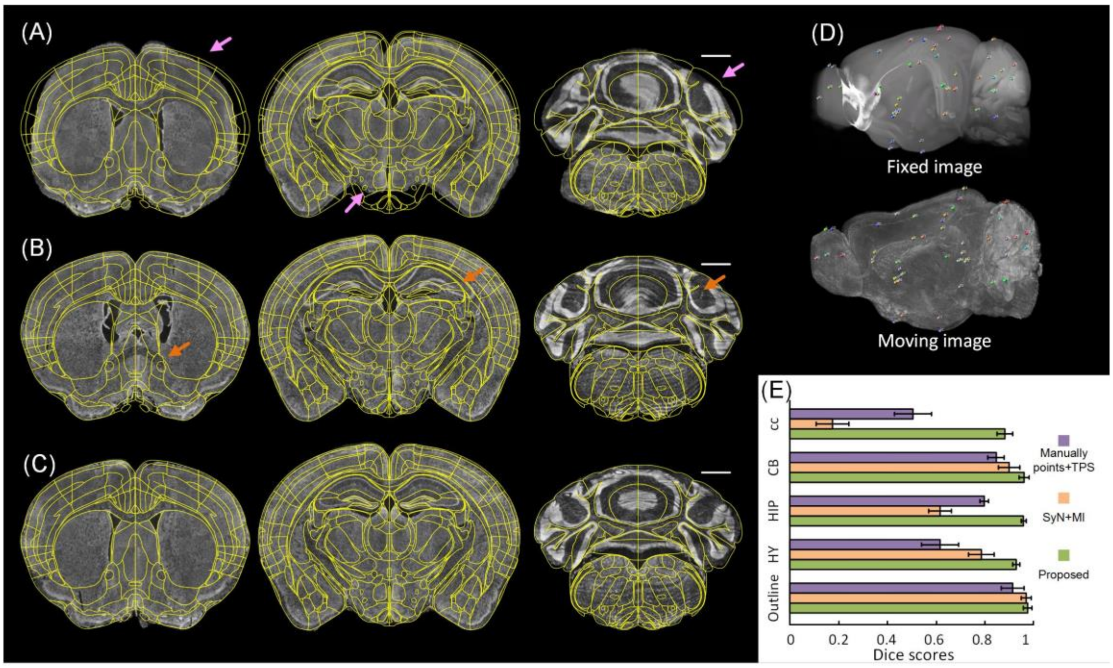

Supplementary Figure 4 Comparisons of different registration methods on the real biological brain dataset

A. The registration results of point-based method (TPS). The inaccurate regions are indicated in purple arrows. **B.** The registration results of gray-level-based method (SyN). The inaccurate regions are indicated in red arrows. **C.** The registration results of proposed BrainsMapi method. **D.** The 3D display of selected points in the fixed and moving datasets, and the points are used in the point-based method of (A). **E.** Quantitative assessments of the above three methods by using the Dice scores. Scale bars: (A, B, C) 2 mm. The reference brain atlas for registration is from the Allen CCF v3, © 2004 Allen Institute for Brain Science. Allen Mouse Brain Atlas. Available from: atlas.brain-map.org.

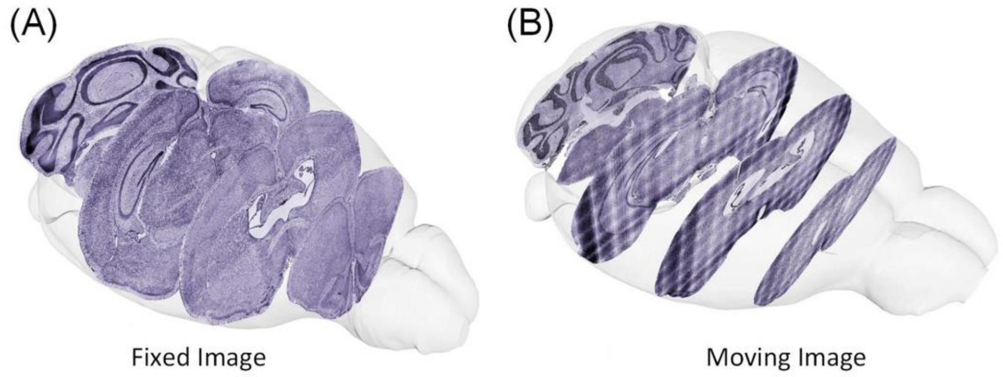

Supplementary Figure 5 The display of specifically selected problematic dataset

Comparisons of 3D rendering outline and coronal planes between normal and specifically selected problematic datasets. **A.** The 3D rendering outline and several coronal sections of Dataset4. **B.** The 3D rendering outline and several coronal sections of Dataset5 with obvious streaks and tears.

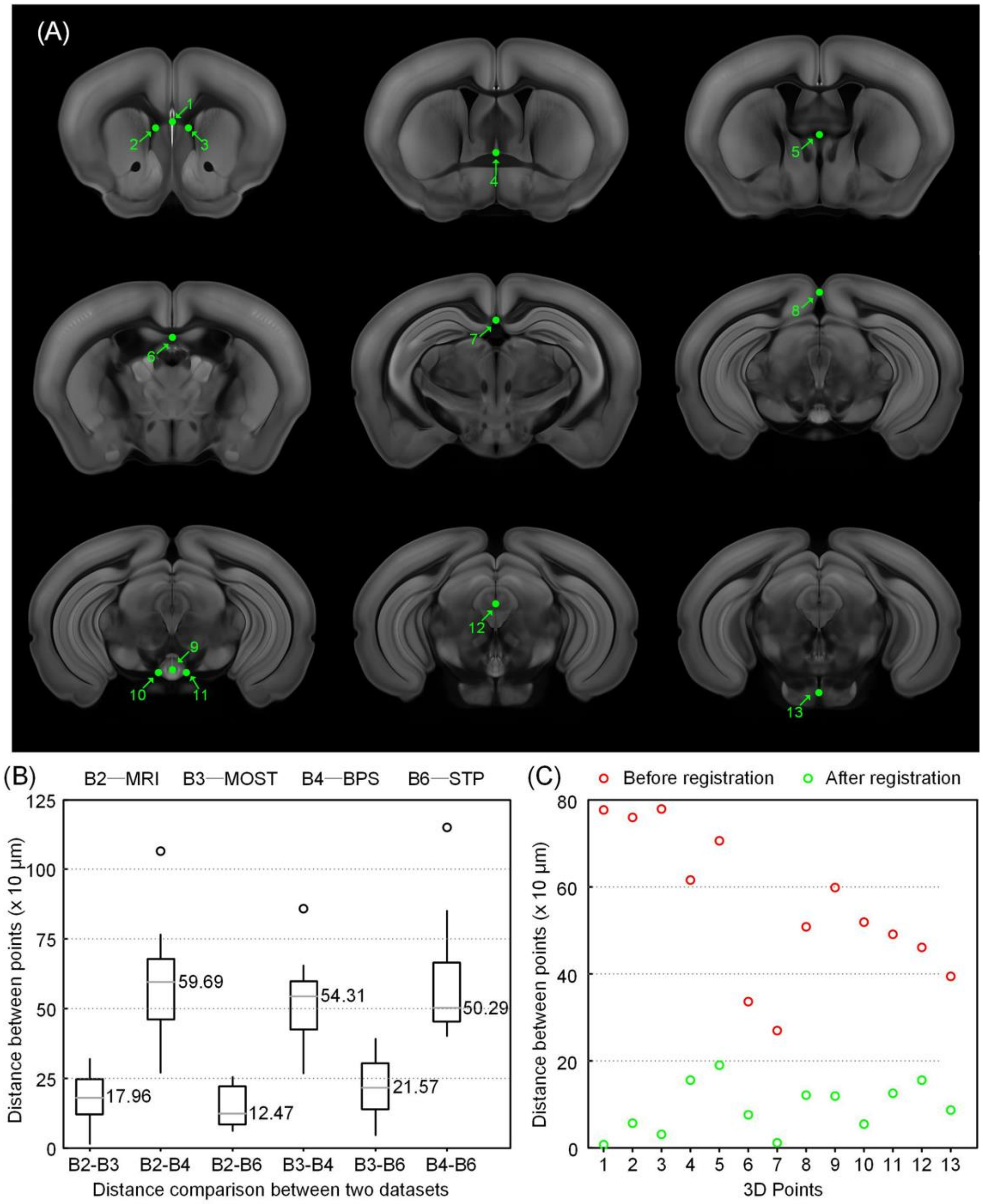

Supplementary Figure 6 The search for large different individuals

A. Thirteen selected feature points in Allen CCFv3. (1) Frontal middle 1 (FM1), (2) Frontal left 2 (FL2), (3) Frontal right 2 (FR2), (4) Crossing of the anterior commissure (AC), (5) Ventricle middle 1 (VM1), (6) Corpus callosum middle (CCM), (7) Hippocampus middle (HM), (8) Cortex middle (KM), (9) Interpeduncular nucleus middle (IP), (10) Interpeduncular nucleus left (IPL), (11) Interpeduncular nucleus right (IPR), (12) Periaqueductal gray middle 3 (PAG3), (13) Pontine nucleus middle (PM).

B. We chose the following datasets of Dataset 2 (MRI), Dataset 3 (Nissl stained, MOST), Dataset 4 (PI stained, BPS) and Dataset 6 (STP) for testing. We selected these 13 feature points in each dataset, and then calculated the distance of these 13 pairs of feature points in each pair of datasets. As shown in (B), that the largest median of the feature point pairs in four datasets is B2-B4 (596.9 μm). **C.** In particular, we have given the distance changes of 13 feature point pairs before and after registration. The red and green circles indicate the distance of the feature point pairs before and after registration respectively. Allen CCF v3, © 2004 Allen Institute for Brain Science. Allen Mouse Brain Atlas. Available from: atlas.brain-map.org.

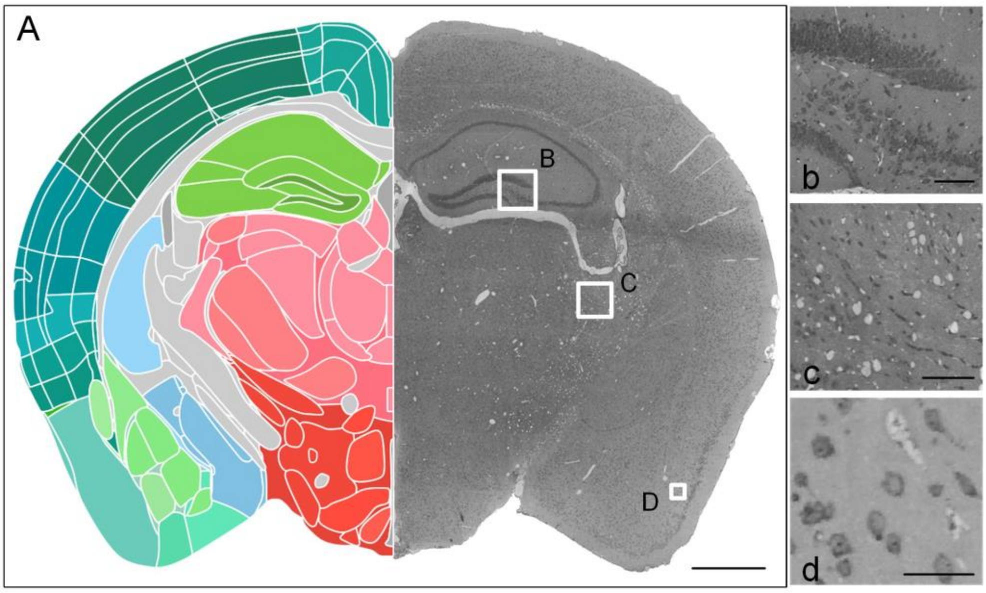

Supplementary Figure 7 Registration effects for a Nissl staining dataset at single-cell resolution

A. A coronal section of Nissl staining dataset after registration, the left half is the Allen CCFv3, and right half is the registered image. **B-D.** The enlarged views of white boxes corresponding to **A**. Scale bars: A 1 mm, (b, c) 100 μm, d 50 μm. Allen CCF v3, © 2004 Allen Institute for Brain Science. Allen Mouse Brain Atlas. Available from: atlas.brain-map.org.

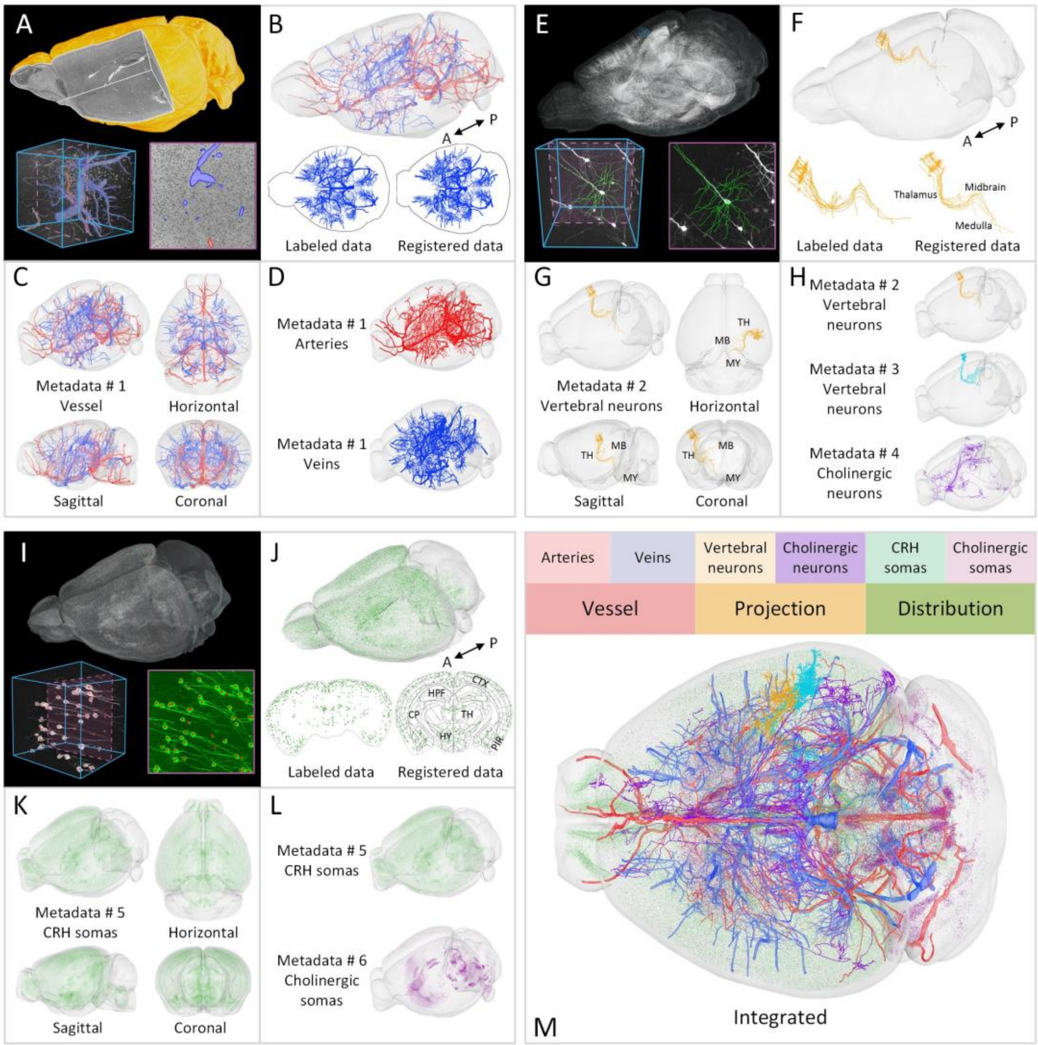

Supplementary Figure 8 Registration effects for existing metadata

A. The 3D rendering of Nissl-stained mouse brain (top), the 3D and 2D reconstructions of the blood vessel (bottom). **B.** Top, the reconstructions of 3D whole-brain arteriovenous blood vessel (arteries: red, veins: blue); Bottom, a comparison of the veins before and after registration in horizontal plane. **C.** Three anatomical views of the arteriovenous vessels after registration (horizontal, sagittal, coronal). **D.** 3D reconstructions of arteriovenous vessels after registration. **E.** The 3D rendering of the GFP channel of Thy1-GFP M-line transgenic mice (top), the 3D and 2D vectorized neurons (bottom). **F.** Top, the 3D reconstruction of vertebral neurons and mouse outline; Bottom, the 3D reconstructions of vertebral neurons before and after registration. **G.** Three anatomical views of the vertebral neurons after registration (horizontal, sagittal,

coronal). **H.** The 3D reconstructions of registered vectorized neurons of multiple datasets. **I.** The 3D rendering of CRH-Cre transgenic mice (top), the 3D and 2D cell bodies rending results (bottom). **J.** Top, the 3D reconstructions of cell bodies in whole-brain; bottom, coronal sections of the cell bodies before and after registration. **K.** Three anatomical views of the registered cell bodies (horizontal, sagittal, coronal). **L.** The 3D reconstructions of registered cell bodies of multiple datasets. **M.** Vessel, barrel cortical neurons, cholinergic neurons, CRH cell bodies, and cholinergic neuronal cell bodies are integrated in the framework of the standard brain space (Allen CCFv3). Allen CCF v3, © 2004 Allen Institute for Brain Science. Allen Mouse Brain Atlas. Available from: atlas.brain-map.org.

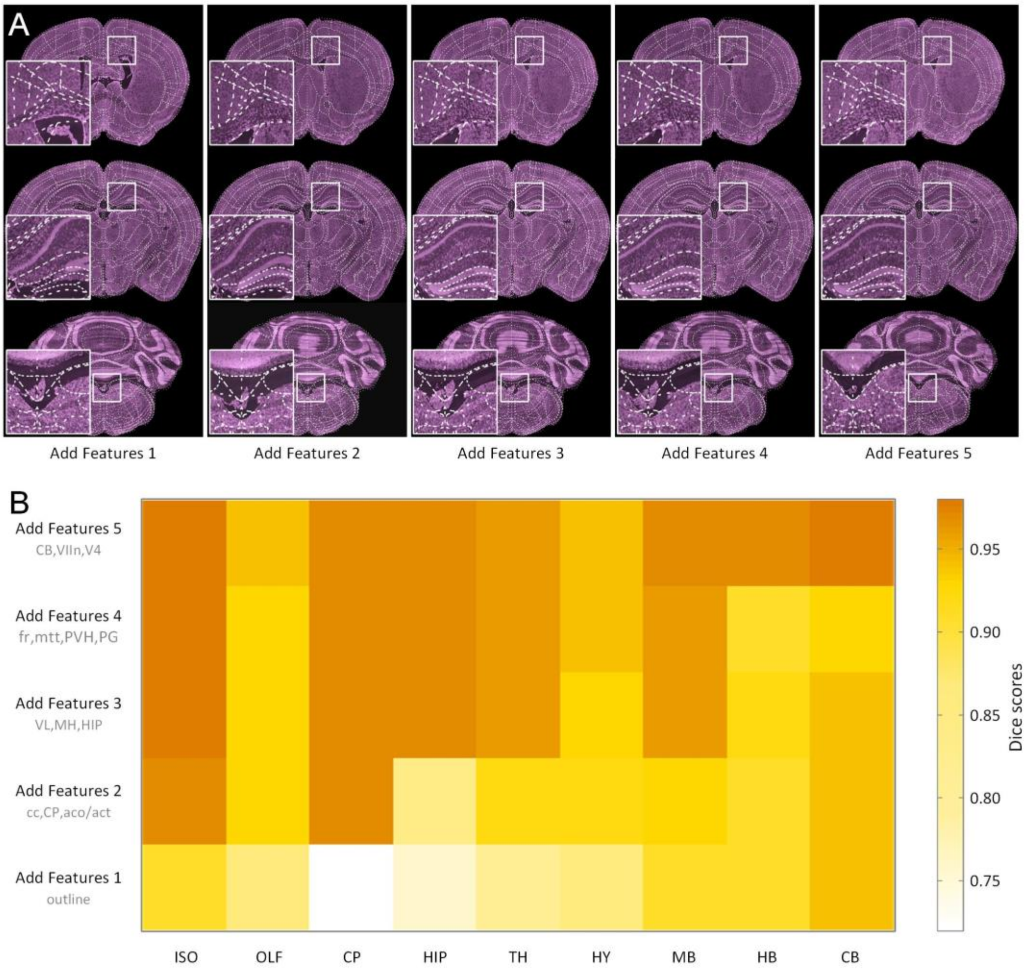

Supplementary Figure 9 The analysis between number of selected features and registration accuracy.

We group these fourteen features into five groups from the olfactory bulb to hindbrain direction of mouse brain, and test the registration performance with continuously adding these five groups of features. **A.** Three coronal sections are presented to show the registration results. With continuous addition of features, the registration effects are improved. **B.** A heat map showing that the Dice scores of nine representative brain regions changes with the addition of features. The Dice scores of these nine brain regions are approximately to 0.95 after adding all the features.

Supplementary Table 1 Detail information of whole brain imaged datasets and metadata

Dataset	Type	Raw Data size	Volume (pixel^3)	Resolution (μm^3)	Description
Dataset1	Reference	1.12 GB	1140 × 800 × 1320	10 × 10 × 10	Allen CCFv3 average brain.
Dataset2	MRI dataset	256 MB	512 × 512 × 1024	21.5 × 21.5 × 21.5	Waxholm single brain T2*.
Dataset3	Nissl stained dataset	4.02 TB	25200 × 14820 × 11843	0.35 × 0.35 × 1	Imaging with MOST system.
Dataset4	PI stained dataset	2.7 TB	28452 × 21866 × 4834	0.32 × 0.32 × 2	Imaging with BPS system.
Dataset5	PI stained dataset	6.01 TB	31223 × 20410 × 10732	0.32 × 0.32 × 1	Imaging with BPS system, specifically selected problematic datasets with stripes and tears.
Dataset6	STP dataset	0.90 GB	1080 × 732 × 1220	12.5 × 12.5 × 12.5	Imaging with STP microscope.
Metadata1	Digital vessel datasets	3.8 GB	1620 × 1103 × 2263	5 × 5 × 5	Segmented vessels from Dataset3.
Metadata2	Vertebral neuron fibers	—	—	—	Neurons traced from Dataset4.
Metadata3	Vertebral neuron fibers	—	—	—	Neurons traced from Dataset5
Metadata4	Cholinergic neuron fibers	—	—	—	From [33]
Metadata5	Cholinergic cell bodies	—	—	—	From [33]
Metadata6	Corticotropin-releasing hormone cell bodies	—	—	—	From [31]

Supplementary Table 2 Detail information of the performance data

Resolution (μm^3)		81.9	41.0	20.5	15.0	10.2	5.1	2.6	1.3	0.64	0.32
Data size (GB)		0.001	0.009	0.073	0.178	0.58	4.66	37.32	298.5	2388	19106
Transform time(h)		---	---	0.01	0.02	0.02	0.05	0.13	0.33	2.78	24.83
Memory Usage (GB)	ITK	0.65	3.16	23.28	54.62	---	---	---	---	---	---
	ANTs	1.26	10.47	85.58	---	---	---	---	---	---	---
	Proposed	---	---	0.25	0.31	2.56	11.52	11.78	12.03	13.01	13.69
